# Training Plan for the Continuity of Non-Presential Education in Six Peruvian Universities during COVID-19

**DOI:** 10.3390/ijerph19031562

**Published:** 2022-01-29

**Authors:** Lourdes Pérez-Sánchez, Silvia Lavandera-Ponce, Begoña Mora-Jaureguialde, Ana María Martín-Cuadrado

**Affiliations:** 1 Department of Didactics, School Organisation and Special Didactics, Universidad Nacional de Educación a Distancia (UNED), 28040 Madrid, Spain; lperezsanchez@edu.uned.es; 2 Excellence Centre for Teaching and Learning, University of Engineering and Technology (UTEC), Lima 15063, Peru; slavandera@utec.edu.pe; 3 Department of Pedagogy, Universidad de Huelva (UHU), 21007 Huelva, Spain; bego.mora@dedu.uhu.es

**Keywords:** higher education institutions, pandemic, digital competence, techno-pedagogical design, virtual training, tutorial support

## Abstract

This article shows the response offered by the UTEC-UNED-TECSUP Consortium to six Peruvian public (national) universities aimed at strengthening the digital competences of their communities, made up of managers, teachers, students, and support technicians. The contextual and situational diagnosis, which covered organizational, technological, and competence dimensions, revealed a series of cross-cutting needs related to technological skills that prevented the training or mobilization of the digital competences necessary for progress in the other dimensions under study. The response was an online training plan, consisting of three training programs and eighty-three courses. The pedagogical strategy was based on the scaling of competence achievements that ended with problem-solving and applications in daily activities. The accompaniment was carried out through virtual tutorials, distributed via synchronous and asynchronous sessions. In total, 5034 were involved—347 were teachers and managers, 4932 were students, and 25 were technical staff. The pedagogical and socio-emotional limitations of the university community, as well as the scarcity of technological resources and poor connectivity, meant that the plan was only partially implemented. Moreover, the short and intense period of development to which the universities were subjected was also a factor.

## 1. Introduction

In March 2020, the World Health Organization [WHO] declared the COVID-19 outbreak a pandemic, urging states around the world to take immediate action to ensure the population’s wellbeing.

Education was one of the most affected sectors globally and locally. Some universities were able to replace face-to-face learning with distance learning quickly. Others needed to develop solutions that ensured the continuity of technology-mediated non-face-to-face education services [1]. “Most universities have had to rethink what they were doing and look for strategies that allow them to continue with their educational work, but not face-to-face” ([2], p. 1).

In Latin America and the Caribbean, “the higher education sector was also unprepared for a disruption such as the one brought about by the COVID-19 pandemic” ([3], p. 23). “In some Latin American countries there were simply no classes, such as in whole areas of Brazil, Venezuela, Honduras, and Paraguay, while in others such Chile, Colombia, and Uruguay, there was progress in spite of the divides towards virtuality” ([2], p. 2).

On average, slightly less than 50% of households in Latin America have Internet access, although it could be assumed that in those with university students it would be slightly higher. The connectivity rates show a wide range of asymmetry between countries. While in Bolivia and El Salvador barely 20% of households are connected, in Argentina and Chile around eight out of ten households have access to the Internet ([1], p. 4).

Connectivity in Latin America represents about 45% of households [2]. Regarding the use of computers as part of this technological divide, different percentages were observed in the Latin American region in 2018, ranging from 70% in Uruguay to 13% in Nicaragua [2].

In the case of Peru, universities decided to postpone the start of the academic cycle to “design their online teaching strategies”; however, “in May 2020, there were still twenty-one universities, eleven public and ten private, which had not yet defined their online teaching starting date” ([4], p. 22). “Although teaching did start, admission processes could not be carried out in most of the national universities, since the dates of the tests coincided with the closure of the educational institutions” ([4], p. 21). Given this situation, it has been essential to strengthen collaborative work between institutions and teachers [5].

The main difference between public (national) and private universities lies in the fact that the former depends on the Peruvian Ministry of Education and the latter on student pensions to ensure its sustainability. Additionally, both are governed by the University Law No. 30220 and by SUNEDU (National Superintendence of University Higher Education), the body in charge of ensuring the quality of the educational offering through the licensing and supervision of this public service. According to INEI (National Institute of Statistics and Informatics), there are 51 national and 89 private universities in Peru, with 339,000 and 752,000 students enrolled, respectively, as of March 2021. Therefore, we are witnessing growth in Peruvian private universities. The data indicate that the total number of students has surpassed one million and that there are 70,000 teachers, most of whom are part-time.

Even though “for some governments COVID-19 was from the beginning a social, economic and political problem, rather than a pedagogical and didactic one” ([6], p. 116), on 16 March 2020, the Peruvian government mandated the closure of universities and the need to adopt emergency measures to guarantee the continuity of non-classroom education services.

On 27 March 2020, the Executive Council Resolution—039-2020 SUNEDU came into force, authorizing the Ministry of Education, through Article 21 of Emergency Decree No. 026-20, to establish regulatory provisions and relevant guidelines for public and private educational institutions under the sector’s jurisdiction in modalities that provide the service using non-face-to-face or remote mechanisms. Similarly, on 1 April of the same year, the guidelines for the continuity of the university education service were approved, highlighting the need to strengthen the digital competences of teachers to guarantee quality in virtual teaching.

The UNED-UTEC-TECSUP Consortium was created within the framework of Call 8 of the Program for Improvement and Relevance of University and Technological Higher Education Services at the National Level (PMESUT).

The Program for the Improvement of the Quality and Relevance of University and Technological Higher Education Services at the National Level (PMESUT) was created between the Government of Peru and the Inter-American Development Bank (IDB) to implement initiatives together with the Ministry of Education to ensure quality university education at the national level. Among the specific objectives stated in its mission are the below points:

- To improve knowledge and information to guide policy decisions aimed at ensuring the quality and relevance of higher education;
- To strengthen the institutional framework of public higher education universities (ESU) and higher technological education (EST) to provide relevant and quality educational services;
- To ensure adequate infrastructure and equipment for public higher education institutions.

As part of these initiatives, there are competitive funds for public universities such as Call 8, framed within the COVID-19 socio-sanitary emergency.

The aim was to contribute to the strengthening and implementation of strategies for the continuity of higher education services in Peru’s public universities. This call was organized into three phases, namely diagnosis, design, and training, as well as techno-pedagogical accompaniment organized into three dimensions, namely: technological, organizational, and digital competences of teachers, students, and technical support staff.

This transition was anticipated, which “can be decomposed into several interconnected dimensions that impact the feasibility and the quality of the distance learning provided, namely the technical infrastructure and accessibility, distance learning competences and pedagogies, and the field of study” ([7], p. 24). Likewise, it is necessary to understand the main concerns of teachers and students in relation to the changing technology, which is conducive to the integration of digital literacy in the teaching–learning process [8].

**Figure 1:**
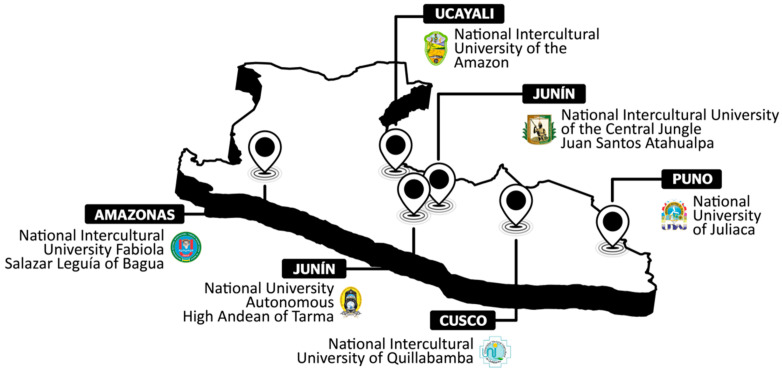
Map of the six Peruvian public universities. Note: Adapted from ([10], p. 2).

This intervention was conducted in six national universities located in the Peruvian highlands and jungle areas, four of which are recognized as intercultural areas (Figure 1). The intercultural policies developed for the incorporation of indigenous collectives and ethnic groups in higher education have not achieved the goal of avoiding cultural inequality [9]. Only a few cultural extension projects in the territories have made visible the greatness of indigenous cultures and their possible contributions to the dominant culture.

Some basic principles were taken into account to design and implement the training plan in intercultural universities, namely pluralism, tolerance, intercultural dialogue, and inclusion; rejection of violence, intolerance, and discrimination; public and professional ethics; recognition and strengthening of the country’s cultural and linguistic diversity; the construction of a democratic coexistence; and equal rights, without discrimination or renouncing their customs and values and respect for cultural differences. It was not easy to create adequate conditions to deal with cultural diversity and intercultural communication, as well as to respect bilingualism.

To address these differences, the training plan was customized, respecting the idiosyncrasies of the university community. Some unique actions were implemented, namely communication with the universities was carried out in the main language, certain model courses on indigenous languages were selected and virtualized, and the universities’ virtual communities served as information and communication channels to strengthen intercultural values and promote the ethnic richness of indigenous peoples. The aim was to highlight the historical roots of the cultures from which today’s society originated [10].

This article describes the training stage, which was based on the following objectives:

EO1. To develop a training plan that integrates the university staff and responds to the techno-pedagogical needs;
EO2. To design and implement the training plan and its follow-up phase.

In this sense, a training plan was organized and implemented. It lasted 90 days and was based on a specific diagnosis of the target audience. An integrative learning path was drawn up to respond to real present and future challenges in the adaptation from face-to-face to non-face-to-face classes. From the outset, the methodological principles underpinning distance learning were considered, ranging from the singularity and roles of the figures involved in the design and development of the plan (pedagogical support, technical support, and virtual support), the beneficiary actors (target audience), and the alignment of the curricular elements, including the objectives, content, activities, assessment, resources, and time involved. In addition, the idea of learning through research and reflection was pursued [11] as the basis of competency-based training.

The needs obtained were categorized in relation to each stakeholder, prioritizing courses of accelerated need for all those involved on a compulsory basis. In addition, a series of more reflective and in-depth training was provided to address specific needs and interests identified in each context.

The training path was developed by combining synchronous training sessions in WebEx and asynchronous training sessions in Moodle. The follow-up was conducted in two periods—firstly during the training implementation and secondly during the third phase of the consultancy, called the techno-pedagogical accompaniment.

## 2. Methodological Approach: The Emergency Training Plan

The assignment from the Ministry of Higher University Education (MINEDU), through PMESUT to the consortium was to support the continuity of the educational service through various strategies. For this, an advisory plan was structured in three stages: diagnosis, training, and techno-pedagogical support in virtualization processes.

The training plan resulting from the second stage emerged after the needs analysis report. This served as a pedagogical tool, provoking changes in the teaching–learning methodological processes, as well as in virtual training model for the future. Likewise, it was key to develop the third stage, since the mentoring process followed implied moments of teacher training and the programmed courses served to respond to conjunctural needs, in addition to structural needs [12].

**Figure 2:**
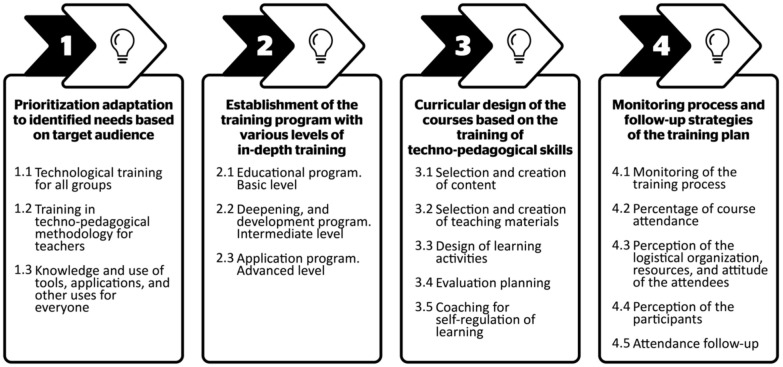
Representative milestones of the emergency training plan. Note. Scheme designed based on the information presented by Martin-Cuadrado et al. Adapted from [10].

Four milestones marked the design and development of the training plan (Figure 2). These described typical actions inspired by a techno-pedagogical design, “whose objective not only seeks to give a comprehensive dimension to the process, but also a more integrative one regarding the use of technology for pedagogical and didactic purposes” ([13], p. 47). The focus was on the diversity of the group encountered, on the typology of the universities, and on the development of programs and courses that were adapted to the needs encountered. Descriptors and indicators were then defined, facilitating the creation of a multi-case working methodology, which was transferable to other similar contexts, given that the six universities were different from each other.

In the following figure (Figure 2) you can see the steps followed to prepare the Training Plan.

### 2.1. Sample

The population was as shown in Table 1.

As the consortium progressed through the advisory process, the “target audience” was conceptualized according to the following parameters:

By definition:
  1. Teachers and managers. Managers and lecturers were differentiated according to the role of responsibility in their institution (academic management, administrative management);
  2. Students. Two categories were established, namely new students and novices, in terms of digital skills.
  3. Technical staff. ICT teams from the universities.
Based on the situation and relationship with the teaching–learning processes:
  1. The role of managers, teachers, and support technicians focused on adapting the educational model and teaching methodology to student learning, from analogue to digital [14,15,16];
  2. The role of students shifted between the training of digital competences and self-regulation of learning, with the aim of autonomously incorporating themselves into distance learning environments [17].

The groups occupied a key space in the educational scenario. According to the pandemic situation and the profile described, they required concrete and specific training.

### 2.2. Initial Needs Analysis

In order to obtain the needs of the different universities, the training plan was implemented based on the results of a simultaneous global diagnosis. This process was conducted over a period of one month and allowed the design of training actions adapted to the requirements of the groups in each university community.

The initial diagnosis was conducted thanks to various information-gathering techniques, such as group and individual interviews (with managers, teaching staff, and students), thematic discussion groups (teaching staff and students), documentary analysis (regulations and academic standards), as well as non-participant observation of virtual spaces and documents.

From all of the information, it was determined that in terms of organization, none of the universities had adapted a protocol for technological support. The user management and response systems were still at the basic level of development, as were the services responsible for academic matters. Regarding monitoring and communication with students, the level was minimal. In the teaching management area, two of the six universities had a high number of hired teachers (30% of the teaching staff), which had an impact on the institutional organization. Regarding the technological dimension, the biggest drawback observed was the lack of connectivity for students. Finally, for the skills dimension, shortcomings were detected in terms of pedagogical and digital knowledge, namely in the range of teachers’ functions both in their digital profiles and in their use of the tools, since they were used little and ineffectively.

A more local diagnosis, which was agreed upon between the consortium and the university authorities, helped to bring the reality of each institution closer together. Once the main training needs were identified, the diagnosis revolved around the following themes:

- Teachers’ and students’ digital skills;
- Management and evaluation processes;
- Methodological and pedagogical strategies in virtual environments;
- Internal quality assurance systems;
- Student reception;
- Reporting in the LMS.

Thanks to the information obtained, it was determined that the six universities were within the ‘emergency’ level at the institutional level [18]. This will mean that greater effort is needed in the transition from analogue to digital institutional structures, as this change is not a choice but a matter of necessity due to the global pandemic. These universities were between the first and second phase of digitization [19].

Subsequently, we identified the needs by target audience, namely for managers, teachers, students, and technicians. As shown in Table 2, the teachers’ needs were related to methodological strategies, evaluation systems, and the use of collaborative tools in virtual contexts. For managers, the needs focused on issues related to the monitoring of activities, the implementation and management of quality assurance systems, and the accompaniment of teaching staff. Students were interested in training in time management, the use of virtual and collaborative tools, and learning how to use Moodle. Finally, the group with the most specific needs was the technical staff, who showed interest in the structural issues of the universities, such as the integration of non-existent systems (the implementation of Moodle), development of recently incorporated tools or tools still at basic use levels (virtual laboratories, management of virtual repositories, licensed tools, integrated video calling systems, remote assistance, incident management, report generator, user support), as well as the development of instructional materials (Table 2).

The last level of training specificity became apparent during the implementation period of the training plan, when each of the participants had to select the courses designed according to their own preferences. The guidance provided by the consultancy to the groups that required it was determinant: for teachers, we mention the association of virtualization activities of subjects and the need-to-know tools to design online forms; for students, the association of tasks as collaborative work activities and the need-to-know collaborative tools to work in teams.

In two of the universities, there was no specific e-learning platform, although G-Suite was used to support the virtualized subjects. One of the cases it corresponded to a large population dispersion and added difficulty in terms of connectivity and technological resources. To bridge this digital divide, both structural and educational efforts were made—the institutions developed a plan to provide devices and data to the students who required them, the number of technical staff was increased, and the consortium drew up a complementary training plan parallel to the general training plan. The content of the parallel training program in these universities with less or no knowledge of Moodle was based on up to six sessions, at a rate of one or two per week, aimed at teaching staff and focusing on the fundamental elements necessary for a proper use of the platform in e-learning. At the same time and focusing on the technical staff in charge of the implementation, development, and management of the tool in the universities, we designed training sessions focused on enabling them to obtain enough skills to be autonomous once the project was completed.

The training plan is explained over four sections, which provide an overview of the process followed by the UTEC-UNED-TECSUP Consortium for its design and development.

### 2.3. Intervention Design and Development: The Training Plan

**Figure 3:**
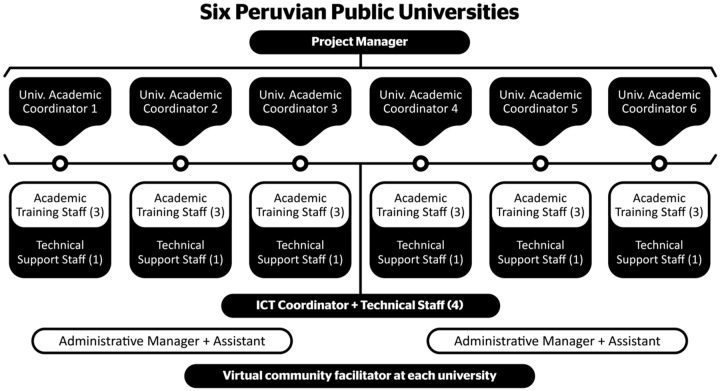
Organization chart for the consortium staff.

There was an emergency staff member in each university, whose mission was to design, develop, and evaluate the training plan. Figure 3 shows the distribution of consortium staff by university.

The training plan was supported by different figures, including academic coordinators, trainers and online tutors, ICT coordinators, support technicians, and the virtual community facilitator.

Table 3 provides a summary of the functions performed by each of them.

During the training plan design and development, the trainer assumed two essential roles, that of a teacher and that of a tutor. In terms of design, as a teacher, they were responsible for the development of the virtual course (content curation, learning guides and manuals, design of learning activities, assessment activities, etc.) and its implementation on the platform. In terms of development, the thematic content was presented synchronously to the target audience of the six universities through the WEBEX platform. According to the assigned courses and their typology, this could be between one and three sessions. In their tutorial role, they guided the target audience of the assigned university through Moodle in an asynchronous way. Depending on the type of course assigned, they had to provide corresponding feedback, as well as an evaluation of the program activities. At all times, they served as learning guides. In addition, they had to manage the forums in terms of overcoming doubts, encouraging participation, answering e-mails, and other tasks. Four courses designed by each trainer, assigned by the academic coordinator. Moreover, as tutor, they were responsible for the guidance of one-third of the courses taught at the assigned university [20].

The roles of the online trainers and tutors were those of a [21] process designer and manager, information and resource provider, learning motivator and facilitator, personal counsellor, facilitator and group facilitator, supervisor, and evaluator. The online trainers were assigned specific roles [22], as opposed to the trainers in face-to-face environments. The online tutorial activities were classified according to phases or moments (initiation, follow-up, and evaluation of the virtual course) [23].

The milestones in the process are shown in Figure 1. The highlights are listed below.

#### 2.3.1. Definition of Training Programs and Courses

The first step, following the needs diagnosis, was to prioritize and synthesize the most basic needs, as follows: training in the virtual teaching methodology; training to lay the foundations on which to build higher quality training courses and applications, tools, and utilities to create digital educational resources and content.

On this basis, we defined three programs and eighty-three courses that made up the training plan. The design of the programs was based on their objective and level of development, considering both their general and applicative nature, as well as the achievement level to be reached in terms of skills acquired by participants. Regarding the course definition with specific topics, we considered the needs of the target audience as listed below.

For managers and teachers:

- Informative program: Basic courses.
- In-depth or development program: General courses.
- Application program: Specific courses.

For students:

- Informative program: Basic courses. Welcome and Initiation courses.
- Application program: Specific courses.

For support technicians:

- In-depth or development program: General courses.
- Application programs: Specific courses.

The goals pursued in each program and the respective courses are outlined below [12].

- Informative program: Basic training on fundamental concepts related to the virtual education methodology, creation of online training actions, and how to transfer face-to-face tasks to virtual tasks. Two-hour course [24].
- In-depth development program: Intermediate acquisition of theoretical and methodological aspects and pedagogical–didactic application of tools and different types of resources (collaboration, communication, tutoring, assessment, etc.). Ten-hour course [25].
- Application program: Advanced proficiency in different applications, tools, and practical utilities from different perspectives according to groups (teachers, students, and technical staff). Fifteen-hour course [26].

During the planning of the training plan, we considered the main elements of any plan, including the themes, groups, development level, achievement levels, and objectives, adapting it to the groups involved in the process.

#### 2.3.2. Course Design: Structure and Working Documents

**Figure 4:**
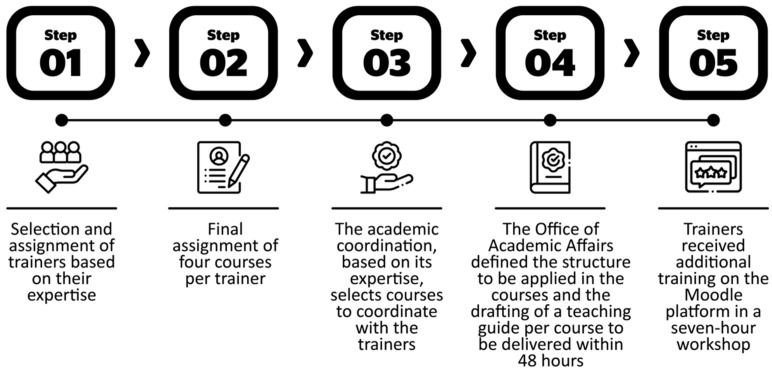
Trainer distribution in the courses.

Once the programs and courses were defined, we set up a process to design and implement them on the platform and then reviewed them. The 83 courses were distributed among the 18 trainers (Figure 4).

During the course development process, there was a peer review, considered as a double or cross-review performed by the academic coordinator with expertise in the subject and the academic coordinator responsible for the trainer. Both reviewed the courses and the basic materials (didactic guides and manuals created). After approval, the trainers proceeded to implement the course on the platform.

##### Framework for Creating E-Learning Courses. Key Elements That Make Up the Course

**Figure 5:**
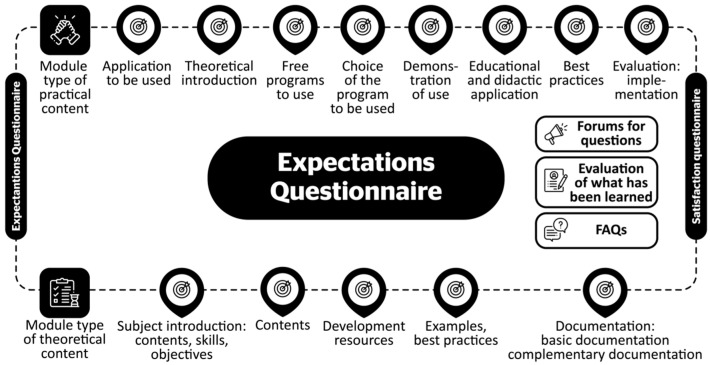
Virtual course structure.

Academic coordinators provided trainers with a structure for course design, as well as to develop the didactic guide (Figure 5). Depending on the type of program to which it belonged, being either theoretical (basic and general courses) or practical (technological application), the evaluations were adapted or complementary resources were added.

This format is completed by teaching materials, presented in different formats and forms, specific to each course according to subject matter and typology [13,27]: text documents for basic content; multimedia documents (presentations, videos, video tutorials, podcasts, etc.); videoconferences; web resources (thematic portals, websites, repositories, databases, etc.); compilations of resources, bibliographic references, electronic references, and additional resources for consultation; technological applications, tools, and programs and guides and manuals for monitoring them.

This diversity offered many possibilities to access the content, with the aim of motivating the learners. In addition, it allowed the use of a wide range of resources and spaces, favoring direct learning, i.e., learning by doing [11,28,29].

The sequence of steps, typology of materials, content, and formats followed a techno-pedagogical design methodology [27], in which several actions were considered:

- Presentation of content in different media and formats, distributed with modules organized in a sequential structure;
- Interaction: Forum for doubts and participation to deepen content;
- Production: Evaluation activities to establish basic content and assess the assimilation.

##### Teaching Guide Framework

On the other hand, the teaching guides were considered a key element for participants, as they were basic orientation documents with essential and necessary information needed to follow the course properly [30,31]. The structural components were the coordination and teaching, target audience (managers, teachers, students, or technical support staff), timeline, objectives, learning outcomes, contents, methodology and activities, and evaluation.

In addition, in the specific courses, the trainers had to design a manual on the application or tools covered by the course. This document helped to learn how to use the specific approach technically and to use it under techno-didactic conditions.

The manuals’ basic structure consisted of the following elements: introduction, objectives, prerequisites, didactic uses (resource management guide, summary of the steps to be followed), recommendations, bibliography, and annexes.

##### Chronogram and Timing (Sequenced Start)

Each course had dates, durations, and a timetable for the synchronous sessions. The timetable was organized in sequences, starting with the basic courses, followed by the general courses, and finally the specific courses.

**Figure 6:**
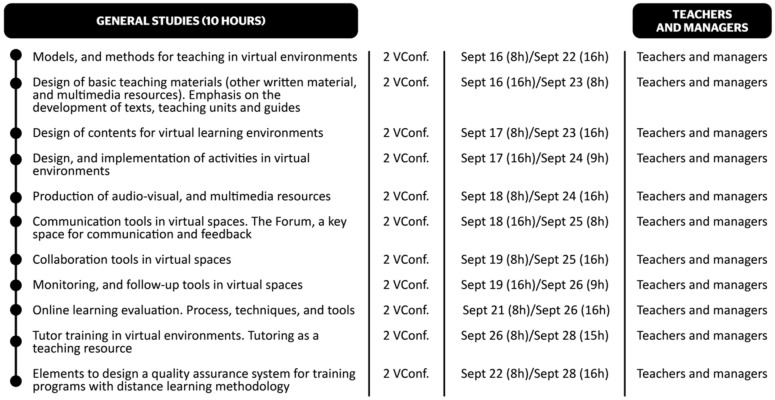
Initial schedule of general courses.

Below, Figure 6 shows an example of the timetable for one of the general course categories, aimed at teachers and managers.

## 3. Results

The most representative results of the above model are presented in the following sections.

### 3.1. Needs by Dimensions

As a first step, we identified the existing needs in each university according to the dimensions: organizational, competence, and technological.

Supplementary Table S1 provides an overview of the percentages achieved in terms of the development levels of the items per dimension.

Considering these data, the training plan had to be complete, ranging from basic to specific knowledge, as it addressed the groups involved in university life, whether in the academic, management, or technical fields. This is how the most important result, the training plan, came about.

### 3.2. Training Plan

As a result of the needs analysis conducted in the diagnosis, a broad, robust, and diverse training plan was designed, adjusted to the university realities that the consortium was dealing with.

We worked to build the thematic categories into which the courses were distributed. Table 4 shows the categorization of the three programs and the eighty-three courses that finally comprised the programs.

The categories were defined to cover general and specific aspects. The aim was to offer complete training on e-learning, its complexity, and its theoretical basis. In addition, e-learning was specified in thematic and group-specific training topics. The full program can be found in Table S2.

### 3.3. Training Scope

Table 5 shows the impact of this training. On average, the initial basic courses that laid the foundations for virtual education (CD-TB and CE-TAS) had the highest participation, followed by the general courses (CD-TG). Subsequently, each person involved, based on their preferences, chose their training route within the 46 courses offered for teachers; for example, as a result, the bulk of the data indicate a higher number of trainings in the practical application courses (CD-TEAP/CD-CA-TEAP/CE-TEAP/CT-TEAP).

### 3.4. Attendance Rates (Synchronous and Asynchronous)

Courses were implemented through synchronous and asynchronous sessions. These were the ways in which the trainers performed their two roles as teachers and tutors. This form of interaction in the courses showed different incidence rates among the groups. Table 6 shows the participation rates in the synchronous and asynchronous sessions according to the target audience.

It is evident that attendance in the synchronous sessions decreased as the course progressed. Participants relied more on asynchrony, having realized that learning could take place asynchronously and autonomously.

### 3.5. Overall Results of the Satisfaction and Expectation Questionnaires

Finally, as an overall evaluation, the answers requested from the target audience, both before and after the training, were collected, regarding their opinions on whether they had a genuine experience of virtual distance learning or training and whether they would recommend this virtual training experience.

Table 7 shows that the number of participants was in line with the number of people trained. In terms of the expectations of the training program, looking at the averages, more than 75% of the respondents had high expectations for the training. These results, while already positive, reached 94.8% in terms of student satisfaction (88.5% for teachers and managers, 58.6% for technicians). All three groups considered the training program to be recommendable, reaching 90% satisfaction in technicians, 93.1% in teachers, and almost 100% in students (99.2%).

## 4. Discussion

The uniqueness of the context of universities resulted in the design of a methodological action research proposal aimed at their improvement [32]. The aim was for the targeted people to be active agents of change and not mere recipients [33]. In this sense, based on knowledge of the characteristics of each institution, adaptations to their idiosyncrasies were made. In order to respond to the first objective, which was the development of an inclusive training plan for the university community, the following questions were posed: What training does each educational community provide? What level of competences does each of the actors in the educational process have? How can the initial situation be improved? What intervention model would be the most appropriate?

The first common response was to use ICT to provide virtual training [34], which would serve as a model for the implementation of virtual education in universities.

In this process, different difficulties appeared related to Internet connections, access to devices, or lack of knowledge for a large part of the university community regarding the teaching and learning process in non-face-to-face environments [10,12]. Other research conducted in Latin American countries during the COVID-19 pandemic showed similar results [1,2,35].

**Figure 7:**
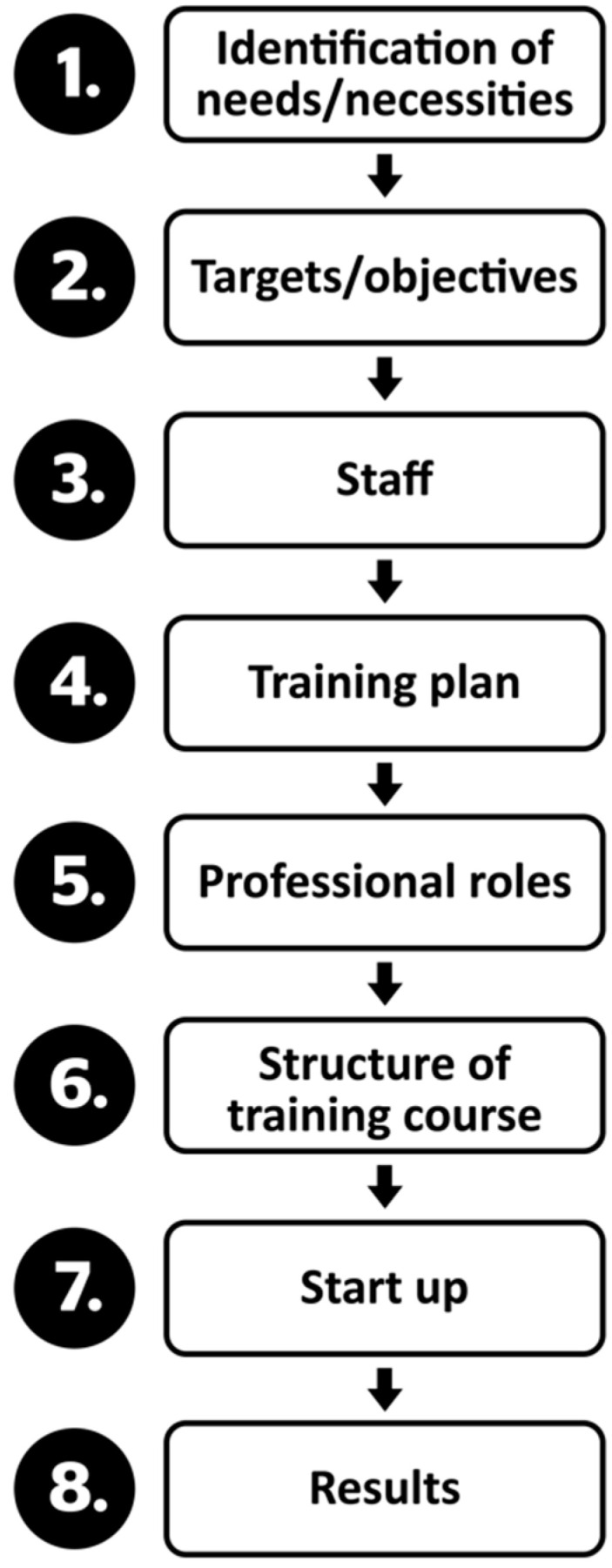
Intervention model.

The research conducted and its subsequent analysis shaped the intervention model from which the training plan was elaborated and eventually developed and implemented by the consortium in the six Peruvian public universities (Figure 7).

This intervention model was based on a reflection of the needs detected within the target population. This action–reflection process made it possible to define the objectives to be addressed by the training plan and which professionals, with different functions, would support the intervention model [11,13,24].

On this basis, we designed a training program integrating different types of training, aimed at each target group, establishing different levels of achievement and covering a wide range of topics, such as an introduction to distance learning, the design and development of digital content, self-regulation competences, internal quality assurance systems, training monitoring processes, and reception of new students.

At the same time as the structures of the different courses and training actions were being shaped, we established a relational plan between the professionals involved in the process and their roles within the framework of the training model, setting the limits between trainers and virtual tutors, as investigated in [23] and described in [21] previously (see Table 3).

Finally, after the implementation of the training, a comparative analysis of participant expectations and satisfaction was conducted (Table 7), whereby two interrelated moments of interest were established, providing constant feedback to each other, the results of which were used to respond to the second objective in this article.

In the first stage, during the implementation of the training, we tried to answer questions such as the following: Does the model fit the predefined needs? Were the results, in terms of participation and involvement of the groups involved, as expected? The findings of the expectation and satisfaction questionnaires completed by the participants, the weekly pedagogical reports by the trainers and tutors, and the technological reports submitted by the support technicians were used for discussion in the weekly meetings held between the consortium and the universities involved. It was necessary to make adjustments to the initial plan on different occasions. The difficulties detected during the implementation were the lack of self-regulation competences for learning in participants, as well as inadequate information regarding the requirements of distance learning, which led to course abandonment and low participation [18]. In addition, the lack of technological resources in homes and the regular use of mobile devices to access and carry out learning activities were identified, without any discrimination. Some studies indicated that the use of mobile devices in student populations is related to social use [36], not academic use. Technical assistance from the university was basic and did not reach the minimum level of demand, which added to the list of difficulties, hindering students and teachers from achieving their expectations. In relation to Latin American universities and the period of transition from face-to-face to non-face-to-face teaching, a previous study confirmed the above-mentioned difficulties [35].

As a result, it was necessary to make temporary adjustments to the training plan, making the delivery of activities more flexible, as well as adding extra training in most cases. In the second moment, at the beginning of the implementation of the consultancy’s third phase, called the techno-pedagogical accompaniment, the follow-up to the training plan’s implementation focused on the impacts and benefits of the plan during the virtualization of subjects or courses. On this occasion, we designed a model of guided support for mentoring between consortium teams and university teachers. According to a study carried out on training procedures with impacts on teachers, the authors pointed out that support and guidance models are significant for novice teachers. In this case, the teachers were new to distance learning [37].

There were difficulties in making progress, which were solved individually or in small groups; the inadequate didactic and digital training of teachers persisted, so whenever possible, they were complemented with personalized, ad hoc training. Vaillant’s contributions on the adaptation of training by Latin American teachers served to strengthen the interpersonal relationships, increase the amount of support provided, and diversify the responses offered, becoming a regular action on the part of the consortium [14]. The short-term impact of the training could be observed at this point. A total of 140 subjects participated, of which 39 did not complete the process in the planned time. A total of 118 teachers were involved.

To close this section, we list the main measures that were considered to alleviate the mentioned difficulties, influencing in a global way phenomena such as drop-out in distance learning ([10], p. 21):

- Measures focused on time management and organization: Planning and alternation of synchronous and asynchronous training sessions in the courses; deferred broadcasting of synchronous sessions; systematic communication about the weekly training schedule through virtual communities;
- Measures for guidance and support to avoid feelings of loneliness and the fear of failure: Involved the tutor and the use of constant feedback [38];
- Measures aimed at strengthening intellectual strategies and study techniques: Curricular documents and materials were designed considering the psycho-pedagogical principles of adult learning;
- Measures to train digital competencies: The courses incorporated training activities that required different levels of academic and digital performance.

## 5. Conclusions

The consortium responded to the needs identified by implementing a training plan for the target audience for three months. The aim was to contribute to the provision of the educational service by ensuring its continuity and facilitating the transition to emergency distance education. Universities were expected to focus their efforts on digital transformation, involving as many representatives of university sectors and areas as possible.

Managers, teachers, and students are the main actors who have had to face the challenge of planning their new roles in response to gaps and changes [2]. The work of managers and teachers has consisted of ensuring the quality of training.

The aim was to prevent teachers from moving from face-to-face to virtual teaching without methodological or curricular adjustments. Pedrò called this “coronateaching” [3]. Similarly, the aim was for students to acquire self-regulatory competences that would allow them to plan and organize their learning according to their original situation and to train and mobilize digital competences that would facilitate the acquisition of learning.

The plan was designed and implemented based on a spiral structure—from basic training to more specific training, with didactic methodology courses coexisting with others of a technical–instrumental nature (ICT tools) [28]. In general, acceptance was satisfactory, even though participation was considered moderate-low. Teachers were the most motivated, followed by students and support technicians. Teachers started with a feeling of uncertainty and dissatisfaction for different reasons—feelings of obligation and overload as they felt they had to attend all the courses offered by the consortium and frustration due to the lack of autonomous learning strategies that would guarantee success during the asynchrony. In addition, they were concerned that access to synchronous sessions was limited and that they did not know how to use WebEx (the videoconferencing tool used by the consortium for the training). Despite the quick response from the consortium, the feeling of insecurity was constant among the participants. Pedagogical, technical, and social shortcomings led to emotional limitations that were experienced as threats throughout the process [3].

The training plan proposed a broad, diverse, and complex training structure, capable of responding to the educational needs of the members of the university community (teachers, students, technical staff, and managers). Some additional courses were included to meet the new demands of the educational communities following other observed needs. The idea of dynamic diagnosis was one of the basic pillars in the implementation of the plan [10,12]. The experiences of the learners, the learning by doing research approach, and participant reflection [11] comprised the methodological basis. This can serve as a model for the implementation of distance learning courses, even in emergency situations.

Learning from experience allows the use of small-scale, personalized, and committed training models—training for a few people, which over time reaches many, involving close and direct training focused on personal rather than group needs. The involvement of university managers is important in the selection of the right people, endorsing their participation in the development of institutional projects that bring about change in the core areas of revolutionary processes in terms of digitalization of educational institutions and their communities.

A training experience close to the idea we are discussing can be seen in the monograph that was published in a Brazilian journal [39]. Seven territorial intervention projects designed to address needs within a global context were assessed. Digital competences were at the heart of all of these projects as a first response. The projects arose from a need detected in the workplace and through an online training model, based on a project-based methodology, with a variety of professionals from the Latin American administration having been trained; they in turn will train other people, while at the same time as they have learned to solve everyday problems through situated learning based on active methodological strategies.

## Figures and Tables

**Table 1:** Population sizes of the six universities.

Teachers and Managers	Students	Technicians
347	4932	25

**Table 2:** Identified needs.

Players	Training Needs
Teachers	Learning assessment. Methodological strategies used in virtual environments. Virtual platform use. Collaborative tools. Technology management (virtual tools). Evaluation systems. Virtual context and activities.
Managers	Teacher guidance. Quality assurance systems. Virtuality monitoring.
Students	Moodle. Time management. Collaborative tools. Tool management. Familiarity with virtual education.
Technical staff	Systems integration Virtual, experimental, and remote laboratories; institutional repositories of learning resources and licensed digital tools. Videoconferencing systems with pedagogical tools. Remote assistance. Incident management. Report generation from different sources (dashboard). Advanced management of Moodle (administrator level). Attention to users and elaboration of training materials.

Note. Adapted from [10] (p. 14).

**Table 3:** Key players in the training process and their roles.

	Players	Number	Roles
Pedagogical support	Academic coordinators	6	Design of the curricular structure of programs and courses.
	Trainers	18	Elaboration of the learning guide, contents, and materials; implementation in the LMS; start-up.
	Tutors		Tutoring, monitoring, and evaluation of course participants.
Technical support	ITC coordinator	1	Planning and execution of the proposal for technical assistance in training implementation.
	Support technicians	25	Follow-up of training support; training in the use of the LMS and educational management systems; monitoring of different groups.
Virtual support	Virtual community facilitator.	1	Facilitation, information, and monitoring of virtual spaces.

Note. Adapted from [10] (p. 9).

**Table 4:** Categorization of training plan courses.

Program	Category	Number of Courses	Target Audience
Basic (Informative) courses	What is digitally supported distance learning? What is virtual learning?	4	Teachers and managers
General courses (in-depth and developmental)	Management of virtual environments in user distance learning, teaching profile.	11	Teachers and managers
Specific courses (practical application)	Teaching models and methods in virtual environments.	6	Teachers
	Design of basic didactic materials.	2	
	Content design for virtual learning environments. Resources used. *	7	
	Production of audio-visual and multimedia materials. *	6	
	Virtual laboratories.	1	
	Design and implementation of activities in virtual environments.	2	
	Communication tools in virtual environments.	5	
	Collaboration tools in virtual environments.	7	
	Follow-up and monitoring tools.	4	
	Evaluation through virtual spaces.	2	
	Tools for academic integrity.	4	
Open courses	Induction courses.	4	Students
	Courses at the beginning of the course.	4	
Specific practical application courses	Application-based courses.	3	Students
Technical courses	Protocols for follow-up monitoring.	11	Technical support staff

Note. * These two categories were common to teachers and students. Courses marked with an asterisk are specific to both teachers and students.

**Table 5:** Training distribution by university according to the type of course.

	UNIQ	UNAAT	UNAJ	UNIA	UNIFSLB	UNISCJSA
CD-TB	17	20	173	58	8	14
CD-TG	16	19	391	41	18	21
CD-TEAP	46	53	740	90	17	70
CD-CA-TEAP	54	18	268	44	19	36
CE-TAS	50	0	11	9	4	3
CE-TEAP	5	0	12	6	0	2
CT-TEAP	16	0	10	5	0	0

Note. CD-TB: Courses for Teachers—Basic (4 courses). CD-TG: Courses for Teachers—General (11 courses). CD-TEAP: Courses for Teachers—Specific Practical Application (34 courses). CD-CA-TEAP: Courses for Teachers and Students—Specific Practical Application (12 courses). CE-TAS: Courses for Students—Induction and Initial (8 courses). CE-TEAP: Courses for Students—Specific Courses for Practical Application (3 courses). CT-TEAP: Courses for Technicians—Specific Courses for Practical Application (11 courses).

**Table 6:** Overall results for technological interaction (synchronous and asynchronous).

Target Audience	Training	Number of Courses	Asynchronous Participation			Synchronous Participation		
			Invited Participants	Active Participants	Trained Participants	Session 1	Session 2	Session 3
Managers and teachers *	Basic courses	4	351	857	290	495	---	---
	General courses	11	351	1389	506	726	781	---
Teachers	Specific courses	34	345	2177	1016	1175	828	420
Teachers and students	Specific courses	12	5366	9490	440	38	28	6
Students	Induction courses	4	5021	5121	71	352	53	---
	Courses at the beginning of the course	4	5021	2809	5	149	38	---
	Specific courses	3	5021	3581	25	37	14	4
Technical staff	General courses	4	92	53	12	29	11	---
	Specific courses	7	161	82	22	40	14	---

Note. * Teachers and managers. In accounting, there is only a difference when the managers only assume a management role in their institution. If they perform teaching duties, they are all included in the calculation as “teachers” ([10], p. 16).

**Table 7:** Summary of the results from the expectation and satisfaction questionnaires.

	Teachers and Managers					Students					Technicians				
	Expectations		Satisfaction		Recommendable	Expectations		Satisfaction		Recommendable	Expectations		Satisfaction		Recommendable
	n	%	n	%	%	n	%	n	%	%	n	%	n	%	%
UNIQ	85	94	54	76	85	106	98	70	97	100	24	100	14	100	100
UNAAT	71	98	52	94	98	0	*	0	*	*	3	67	0	33	*
UNIA	239	92	174	95	100	100	94	47	90	96	7	100	2	50	100
UNAJ	1233	87	725	100	98	67	54	23	87	100	12	80	10	100	60
UNIFSLB	53	42	58	74	78	2	50	1	100	100	0	*	0	*	*
UNISCJSA	32	88	23	92	100	15	100	5	100	100	10	100	1	100	100
PROMEDIO	1713	83.5	1086	88.5	93.1	290	79.2	146	94.8	99.2	56	75	27	58.6	90

Note. * Asterisks indicate that there is no data; “n” represents the number of persons participating in the surveys.

## Data Availability

This study did not include the collection of analysis data.

## Supplementary Materials

The following supporting information can be downloaded at: https://www.mdpi.com/article/10.3390/ijerph19031562/s1, Table S1: Needs identified by dimension to address non-presential education.; Table S2: Training plan list of programmes and courses.
