# Supplementary material for: Training Plan for the Continuity of Non-Presential Education in Six Peruvian Universities during COVID-19
Source: Int J Environ Res Public Health. 2022 Jan 29;19(3):1562. doi: 10.3390/ijerph19031562 (PMC8835666; doi:10.3390/ijerph19031562)
Supplement: Supplementary file 1 [file ijerph-19-01562-s001.zip › Table S1. Needs identified by dimension to address non-presential education.pdf]

Table S1. Needs identified by dimension to address non-presential education.

Dimension	Identified needs	Development
Organisational	Internal regulations, appropriate education policy	100% b
	Organisational units of administrative functioning	100% b
	Adapted educational model	83% b – 17% a
	Course monitoring and evaluation system or instrument	100% b
	Academic and technological innovation	67% a – 33% b
Technological	Training and capacity building programmes for institutional teams	100% b
	Connectivity and features	50% a – 50% b
	Access to institutional technology equipment	50% a – 50% b
	Teaching-learning platform (existence and management)	83% b – 17% c
	Digital tools and licences for non-classroom education	67% b – 33% a
	Virtual libraries	83% b – 17% a
	Virtual laboratories	100% a
	Remote technical assistance	100% b
	Equipment installation, maintenance, and repair	67% c – 33% b
Competence	Knowledge and practice of distance learning.	67% b – 33% c
	Management of virtual Teaching-Learning environments, platforms, and resources	83% a – 17% b
	Organisation and appropriate use of online documentation	33% a – 17% b – 50% c
	Use of simulation materials and tasks	33% a – 67% b
	Use of LMS and digital tools	17% a – 50% b - 33% c
	Creation of materials and activities for virtual courses.	33% a - 67% b
	Design and development of online assessment processes	50% a - 50% b
	Adequate knowledge of pedagogy and didactics applied to virtual environments.	17% a - 83% b
	Course virtualisation	50% a - 50% b
	Monitoring and evaluation of non-classroom courses.	17% a - 83% b
	Efficient use of digital media	83% b -17% c
	Knowledge of non-face-to-face education processes	17% a/67% b - 17% c
	Self-management in non-classroom-based teaching-learning processes	50% a - 50% b
	Collaborative work in non-face-to-face environments	50% a - 50% b
	Capacity for institutional academic and pedagogical management	17% a – 50% b - 33% c
	Capacity for institutional planning, monitoring and evaluation	50% b - 50% c
	Management strategies and instruments	50% a - 50% c
	Computer systems, hardware, and software management	33% b - 67% c

Note. Needs identified as a percentage based on the number of universities showing this need. Universities involved: 6. Levels observed: a. Lowest development level. Highest need; b. Medium development level. Need for improvement; c. Highest development level. Need for deepening and specialisation.
