# Supplementary material for: Training Plan for the Continuity of Non-Presential Education in Six Peruvian Universities during COVID-19
Source: Int J Environ Res Public Health. 2022 Jan 29;19(3):1562. doi: 10.3390/ijerph19031562 (PMC8835666; doi:10.3390/ijerph19031562)
Supplement: Supplementary file 1 [file ijerph-19-01562-s001.zip › Table S2. Training plan list of programmes and courses.pdf]

TRAINING PLAN
LIST OF PROGRAMMES AND COURSES

BASIC COURSES (2 HOURS) — TEACHERS AND MANAGERS

What is distance learning supported by digital resources? What is e-learning?	1 VConf.	14 sept. 8 h.	Teachers and managers
How do I transform my face-to-face course into a distance/virtual course? General guidelines	1 VConf.	14 sept. 16 h.	Teachers and managers
Basic structure for my virtual course: How do I organise my course in the virtual space?	1 VConf.	15 sept. 8 h.	Teachers and managers
How to adapt what I have learnt to my subject/syllabus	1 VConf.	15 sept. 16 h.	Teachers and managers

GENERAL COURSES (10 HOURS) — TEACHERS AND MANAGERS

Teaching models and methods in virtual environments	2 VConf.	16 sept. 8h. / 22 sept. 16h.	Teachers and managers
Design of basic teaching materials (other written materials, multimedia material).	2 VConf.	16 sept. 16h. / 23 sept. 8h.	Teachers and managers
Emphasis on the development of texts, didactic units - guides.	2 VConf.	17 sept. 8h. / 23 sept. 16h.	Teachers and managers
Content design for virtual learning environments	2 VConf.	17 sept. 16h. / 24 sept. 9h.	Teachers and managers
Activities design and implementation in virtual environments	2 VConf.	18 sept. 8h. / 24 sept. 16h.	Teachers and managers
Production of audiovisual and multimedia resources	2 VConf.	18 sept. 16h. / 25 sept. 8h.	Teachers and managers
Communication tools in virtual spaces. The Forum, a key space for communication and feedback	2 VConf.	19 sept. 8h. / 25 sept. 16 h.	Teachers and managers
Collaboration tools in virtual spaces	2 VConf.	19 sept. 16h. / 26 sept. 9h.	Teachers and managers
Accompaniment and monitoring tools in virtual spaces.	2 VConf.	21 sept. 8h. / 26 sept. 16 h.	Teachers and managers
Evaluation of online learning. Process, techniques, and tools	2 VConf.	26 sept. 8h. / 28 sept. 15 h.	Teachers and managers
Tutor training in virtual environments. Tutoring as a didactic resource.	2 VConf.	22 sept. 8h. / 28 sept. 16 h.	Teachers and managers

SPECIFIC COURSES - PRACTICAL APPLICATION (15 HOURS) — TEACHERS AND STUDENTS

Teaching models and methods in virtual environments

Videoforum	3 VConf.	12 oct. 8h. / 19 oct. 9h. / 26 oct. 16 h.	Teachers

Working through an image	3 VConf.	5 oct. 8h. / 12 oct. 9h. / 21 oct. 8h.	Teachers
Going deeper into a topic	3 VConf.	13 nov. 17 h. / 20 nov. 16 h. / 27 nov. 16h.	Teachers
Case study	3 VConf.	19 oct. 8h. / 26 oct. 9h. / 2 nov. 16 h.	Teachers
Project work	3 VConf.	28 sept. 8 h. / 5 oct. 9h. / 12 oct. 16 h.	Teachers
Flipped Classroom	3 VConf.	21 sept. 9h. / 28 sept. 9h. / 5 oct. 16 h.	Teachers
Design of basic teaching materials			Teachers
Teaching guides	3 VConf.	22 sept. 9h. / 29 sept. 9h. / 6 oct. 16h.	Teachers
Study material-textbook	3 VConf.	29 sept. 8h. / 6 oct. 15h. / 13 oct. 16h.	Teachers
Designing content for virtual learning environments: Resources used			
Presentations: PPT, Powtoon, Genially, Prezi, Canva, Emaze	3 VConf.	30 sept. 8h. / 7 oct. 9 h. / 14 oct. 16 h.	Teachers and students
Infographics: Genially, Piktochart, Canva, Infogram, Easelly, Visme	3 VConf.	4 nov. 8h. / 11 nov. 9 h. / 18 nov. 8h.	Teachers and students
Interactive images: Genially, Justapose, Visme, Thinglink	3 VConf.	23 sept. 9 h. / 30 sept. 9h. / 7 oct. 16h.	Teachers and students
Concept Maps: Mindomo, Glosster, Creately, Coogle, Mindmanager, Mindmap, Cmap Tools,Free Mind Map	3 VConf.	14 oct. 8h. / 21 oct. 9h. / 28 oct. 16 h.	Teachers and students
Interactive content: H5P, Apester, Kahoot, Quizlet	3 VConf.	11 nov. 8h. / 18 nov. 9h. / 25 nov. 8h.	Teachers and students
Blogs (Moodle), Blogger	3 VConf.	7 oct. 17 h. / 13 oct. 15h. / 21 oct. 16h.	Teachers and students
Timelines: Timeline, Sutori, iSpring, OiceTimelin, Visme	3 VConf.	28 oct. 8h. / 4 nov. 9h. / 11 nov. 16 h.	Teachers and students
Audiovisual and multimedia resources production			Teachers and students
Videotutoriales: Jing, Screencast-o-matic, Apowersoft, Ctrl+Alt+G, Vídeos en el FORO de Moodle	3 VConf.	15 oct. 8h. / 22 oct. 9h. / 29 oct. 16 h.	Teachers and students
Animated videos: Moovly, Weebly	3 VConf.	8 oct. 8h. / 15 oct. 9h. / 22 oct. 16 h.	Teachers and students
Youtube channels: crear, subir, editar	3 VConf.	22 oct. 8h. / 29 oct. 9h. / 5 nov. 16h.	Teachers and students
Video editing: Camtasia, OBS Studio	3 VConf.	24 sept. 15 h. / 1 oct. 9 h. / 8 oct. 16h.	Teachers and students
Podcasts	3 VConf.	1 oct. 8h. / 8 oct. 9h. / 15 oct. 16h.	Teachers and students
Interactive whiteboards: Webpaint y Jamboard	3 VConf.	29 oct. 8h. / 5 nov. 9h. / 12 nov. 16h.	Teachers
Virtual labs			
FisQuiweb, PhET, Educaplus, Go-Lab	3 VConf.	6 oct. 17 h. / 13 oct. 9 h. / 20 oct. 16h.	Teachers
Design and implementation of activities in virtual environments			
Activities related to learning objects-moodle	3 VConf.	13 oct. 8h. / 20 oct. 9h. / 27 oct. 16h.	Teachers
Activities related to learning objects-open resources	3 VConf.	20 oct. 8h. / 27 oct. 9h./ 3 nov. 16h.	Teachers
Communication tools in virtual environments			
Forums	3 VConf.	25 sept. 15 h. / 2 oct. 9 h./ 9 oct 16 h.	Teachers
Chats	3 VConf.	2 oct. 16 h. / 9 oct. 15 h. / 16 oct 16 h.	Teachers
E-mail	3 VConf.	9 oct. 8h. / 16 oct. 9h. / 23 oct. 16 h.	Teachers
Videoconferencing: Zoom, Meet, Teams, Skype, ...	3 VConf.	16 oct. 8h. / 23 oct. 9h. / 30 oct. 16 h.	Teachers
WhatsApp	3 VConf.	23 oct. 8h. / 30 oct. 9 h. / 6 nov. 9h.	Teachers
Collaboration tools in virtual environments			
Wikis	3 VConf.	3 oct. 9 h. / 10 oct. 9 h. / 17 oct. 16 h.	Teachers
Glossaries	3 VConf.	10 oct. 8 h. / 17 oct. 9h. / 24 oct. 16h.	Teachers
Portfolios	3 VConf.	26 sept. 10 h. / 3 oct. 9h. / 10 oct. 16h.	Teachers
Google Drive	3 VConf.	7 nov. 8h. / 14 nov. 9h. / 21 nov. 8h.	Teachers

One Drive	3 VConf.	24 oct. 8h. / 31 oct. 9 h. / 7 nov. 16h.	Teachers
Dropbox	3 VConf.	17 oct. 8h. / 24 oct. 9h. / 31 oct. 16h.	Teachers
Bibliographic management tools: Mendeley, Refworks	3 VConf.	31 oct. 8h. / 7 nov. 9h. / 14 nov. 16 h.	Teachers
Tools for accompaniment and monitoring in virtual spaces			
Mentoring, interaction tools	3 VConf.	2 nov. 8h. / 9 nov. 9h. / 16 nov. 8h.	Teachers
Feedback	3 VConf.	9 nov. 8h. / 16 nov. 9h. / 23 nov. 16 h.	Teachers
Rúbricas	3 VConf.	26 oct. 8h. / 2 nov. 9h. / 9 nov. 16 h.	Teachers
Activity reports	3 VConf.	5 nov. 8h. / 12 nov. 9h. / 19 nov. 8h.	Teachers
Evaluation in virtual spaces			
Moodle features-. Questionnaires, surveys…	3 VConf.	30 oct. 16 h. / 6 nov. 9h. / 13 nov. 16h.	Teachers
Google forms, Office 365,…	3 VConf.	27 oct. 8h. / 3 nov. 9h. / 10 nov. 16 h.	Teachers
Tools for academic integrity			
Turnitin	3 VConf.	19 nov. 8h. / 17 nov. 9h. / 24 nov. 8h.	Teachers
Proctoring	3 VConf.	3 nov. 8h. / 10 nov. 9h. / 17 nov. 8h.	Teachers
Gradescope	3 VConf.	6 nov. 8h. / 13 nov. 9h. / 20 nov. 8h.	Teachers
Alternative assessments	3 VConf.	12 nov. 8h. / 19 nov. 9h. / 26 nov. 8h.	Teachers

SYNCHRONOUS OPEN COURSES (2 HOURS) — STUDENTS

Induction courses	2 Editions. Weeks 14 to 19 and 21 to 25 September. It will be possible to do any of the two		
What does the student need to know about your university?	1 VConf.	14 sept. 8 h. / 21 sept. 8h.	Students
What does distance learning supported by digital resources consist of? What is virtual learning?	1 VConf.	14 sept. 16 h. / 21 sept. 16 h.	Students
What tools and resources do you need to master in order to learn in a virtual environment?	1 VConf.	15 sept. 8 h. / 22 sept 8 h.	Students
Zero courses: skills levelling	1 VConf.	15 sept. 16 h. / 22 sept 16 h.	Students
Course at the beginning of the course	2 Editions. Weeks 14 to 19 and 21 to 25 September. It will be possible to do any of the two		
Immersion and understanding my virtual classroom	1 VConf.	16 sept. 8 h. / 23 sept. 16h.	Students
Time management and planning	1 VConf.	16 sept. 16 h. / 23 sept. 17 h.	Students
Tips for accessing my synchronous sessions	1 VConf.	17 sept. 8 h. / 24 sept.. 16h.	Students
Tips for making the most of asynchronous sessions	1 VConf.	17 sept. 16 h. / 24 sept. 17 h.	Students
Specific courses - practical application (15 hours)			
Word processing: How to get the most out of it	3 VConf.	18 sept. / 25 sept. / 02 oct. 8h.	Students
Spreadsheets	3 VConf.	18 sept. / 25 sept. / 02 oct. 17h.	Students
Audiovisual presentations	3 VConf.	18 sept. / 25 sept. / 02 oct. 16 h	Students

FOLLOW-UP AND MONITORING PROTOCOLS COURSES — TECHNICAL SUPPORT STAFF

General courses (10 hours)

Case management via ticketing tool	2 VConf.	14 sept. / 21 sept. / 10h.	Support technicians
Remote assistance support	2 VConf.	15 sept. / 22 sept. / 10h.	Support technicians
Consolidation of frequently asked questions on the help desk	2 VConf.	16 sept. / 23 sept. / 10h.	Support technicians
Analytics Reporting - Videoconferencing systems	2 VConf.	17 sept. / 24 sept. / 10h.	Support technicians

Specific courses - practical application (15 hours)

Moodle for Administrators: Configuring the Moodle platform	2 VConf.	14 sept. / 21 sept. / 15h.	Support technicians
Moodle for Administrators: Backup backups	2 VConf.	14 sept. / 21 sept. / 18h.	Support technicians
Moodle for Administrators: Moodle and course migration	2 VConf.	15 sept. / 22 sept. / 15h.	Support technicians
Moodle for Administrators: Plugins	2 VConf.	16 sept. / 23 sept. / 15h.	Support technicians
Moodle for Administrators: Content Management (repository management)	2 VConf.	17 sept. / 24 sept./ 15h.	Support technicians
Moodle for Administrators: Analytics Reporting	2 VConf.	18 sept. / 25 sept. / 15h.	Support technicians
User support and development of instructional materials	2 VConf.	18 sept. / 25 sept. / 28 sept. / 10h.	Support technicians
